# *catena*-Poly[[diiodidomercury(II)]-μ-nicotine-κ^2^ *N*:*N*′]

**DOI:** 10.1107/S1600536808020904

**Published:** 2008-07-12

**Authors:** Zhengjing Jiang, Guodong Tang, Lude Lu

**Affiliations:** a Key Laboratory for Soft Chemistry and Functional Materials of the Ministry of Education, Nanjing University of Science and Technology, 200 Xiaolingwei, Nanjing 210094, Jiangsu, People’s Republic of China; b Department of Chemistry, Huaiyin Teachers College, Huai’an 223300, Jiangsu, People’s Republic of China

## Abstract

The title polymeric complex, [HgI_2_(C_10_H_14_N_2_)]_*n*_, was prepared from a solution of nicotine, mercury(II) iodide and 4-cyano­pyridine in dimethyl­formamide. Each nicotine mol­ecule is bonded to two Hg atoms, one through the pyrrolidine N atom and the other through the pyridine N atom, forming infinite zigzag polymeric chains. The coordin­ation around mercury is completed by two iodide ligands, resulting in a distorted tetra­hedral arrangement.

## Related literature

For related literature, see: Udupa & Krebs, (1980 ▶); Meyer *et al.*, (2006 ▶); Haendler, (1990 ▶). 
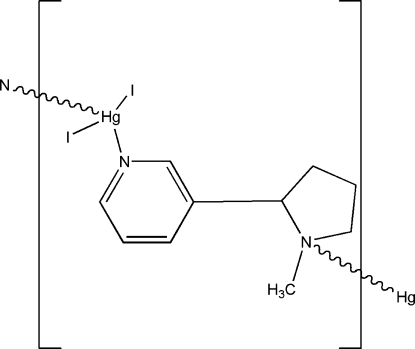


## Experimental

### 

#### Crystal data

[HgI_2_(C_10_H_14_N_2_)]
*M* *_r_* = 616.62
Orthorhombic, 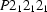

*a* = 7.7171 (2) Å
*b* = 11.1548 (3) Å
*c* = 15.9646 (4) Å
*V* = 1374.28 (6) Å^3^
*Z* = 4
Mo *K*α radiation
μ = 15.67 mm^−1^
*T* = 123 (2) K
0.20 × 0.16 × 0.12 mm

#### Data collection

Bruker SMART APEXII CCD diffractometer
Absorption correction: multi-scan (*SADABS*; Bruker, 2000 ▶) *T*_min_ = 0.062, *T*_max_ = 0.15
6248 measured reflections
2344 independent reflections
2274 reflections with *I* > 2σ(*I*)
*R*_int_ = 0.030

#### Refinement

*R*[*F* ^2^ > 2σ(*F* ^2^)] = 0.023
*wR*(*F* ^2^) = 0.049
*S* = 0.92
2344 reflections
137 parameters
6 restraints
H-atom parameters constrained
Δρ_max_ = 0.89 e Å^−3^
Δρ_min_ = −1.31 e Å^−3^
Absolute structure: Flack (1983 ▶), 852 Friedel pairs
Flack parameter: 0.033 (5)

### 

Data collection: *APEX2* (Bruker, 2004 ▶); cell refinement: *SAINT* (Bruker, 2004 ▶); data reduction: *SAINT*; program(s) used to solve structure: *SHELXS97* (Sheldrick, 2008 ▶); program(s) used to refine structure: *SHELXL97* (Sheldrick, 2008 ▶); molecular graphics: *SHELXL97*; software used to prepare material for publication: *SHELXL97* and *PLATON* (Spek, 2003 ▶).

## Supplementary Material

Crystal structure: contains datablocks I, global. DOI: 10.1107/S1600536808020904/hg2420sup1.cif

Structure factors: contains datablocks I. DOI: 10.1107/S1600536808020904/hg2420Isup2.hkl

Additional supplementary materials:  crystallographic information; 3D view; checkCIF report

## Figures and Tables

**Fig. 1.**
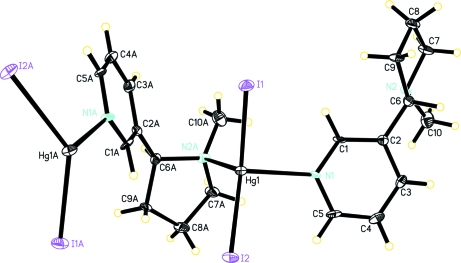
Molecular structure of the title compound, with atom labels and 50% probability displacement ellipsoids. All H atoms have been omitted. Symmetry transformations: A is -x+1/2, -y, z+1/2.

## supplementary crystallographic information

### Comment

Numerous complexes of nicotine [3-(1-methyl-2-pyrrolidinyl)pyridine] have been reported to form molecular complexes and polycomplexes with metals (Udupa & Krebs, 1980; Meyer *et al.*, 2006; Haendler, 1990). However, the crystal structure of the polycomplex, di-iodido(nicotine)mercury(II), has not been reported so far. In order to further explore the structural chemistry of nicotine complexes, we synthesized and determined the structure of the title compound (I).

As illustrated in Fig. 1, each nicotine molecule in (I) is coordinated to two adjacent mercury atoms, one through the pyrrolidine nitrogen (Hg—N 2.428 (7) Å) and the other through the pyridine nitrogen (Hg—N 2.454 (5) Å), forming zigzag polymeric chains. The coordination around mercury is completed by two iodide ligands (Hg—I 2.6819 (6) and 2.6536 (5) Å), resulting in a distorted tetrahedral arrangement. In addition, the absolute configurations of C6 and N2 can be given as *S* (*S*-nicotine was used as a starting material). No notable interactions were found between polymeric chains.

Examples of closely related compounds containing nicotine ligands include a mercury(II) chain polymer (Udupa & Krebs, 1980), a helical silver(I) coordination polymer (Meyer *et al.*, 2006) and a chloride-nicotine copper(II) complex (Haendler, 1990).

### Experimental

HgI_2_ (454 mg,1 mmol) was added to a solution of 4-cyanopyridine (104 mg,1 mmol) in dmf (5 ml). The resulting mixture was stirred for about 10 min after which an white precipitate formed. *S*-Nicotine (3 ml) was then added dropwise to the reaction mixture and stirring was continued, during which time the precipitate changed its colour, giving a flesh colored precipitate. The precipitate was washed with ethanol and vacuum dried. Yield: 0.435 g, 70% (based on HgI_2_used). The compound (100 mg) was dissolved in dmf (5 ml), the resulting solution filtered and the light-yellow filtrate transfered into a test tube and *i*-PrOH (10 ml) was carefully laid on the surface of the filtrate. Light-yellow block crystals were obtained after 15 days. Analysis: Found: C 34.52, H 3.90, N 7.90%; Calculated for C_10_H_14_HgI_2_N_2_: C 34.35, H 4.04, N 8.01%.

### Refinement

H atoms were positioned geometrically and refined using a riding model, with C—H = 0.95–1.00 Å and with *U*_iso_(H) = 1.2 (1.5 for methyl groups) times *U*_eq_(C).

### Crystal data

**Table:**

[HgI_2_(C_10_H_14_N_2_)]	*F*_000_ = 1096
*M**_r_* = 616.62	*D*_x_ = 2.980 Mg m^−^^3^
Orthorhombic, *P*2_1_2_1_2_1_	Mo *K*α radiation λ = 0.71073 Å
Hall symbol: P 2ac 2ab	Cell parameters from 2274 reflections
*a* = 7.7171 (2) Å	θ = 2.1–26.4º
*b* = 11.1548 (3) Å	µ = 15.67 mm^−^^1^
*c* = 15.9646 (4) Å	*T* = 123 (2) K
*V* = 1374.28 (6) Å^3^	Block, light-yellow
*Z* = 4	0.20 × 0.16 × 0.12 mm

### Data collection

**Table:**

Bruker SMART APEX CCD diffractometer	2344 independent reflections
Radiation source: fine-focus sealed tube	2274 reflections with *I* > 2σ(*I*)
Monochromator: graphite	*R*_int_ = 0.030
*T* = 123(2) K	θ_max_ = 25.5º
φ and ω scans	θ_min_ = 2.2º
Absorption correction: multi-scan(SADABS; Bruker, 2000)	*h* = −9→6
*T*_min_ = 0.062, *T*_max_ = 0.15	*k* = −13→11
6248 measured reflections	*l* = −19→15

### Refinement

**Table:**

Refinement on *F*^2^	Hydrogen site location: inferred from neighbouring sites
Least-squares matrix: full	H-atom parameters constrained
*R*[*F*^2^ > 2σ(*F*^2^)] = 0.023	*w* = 1/[σ^2^(*F*_o_^2^)] where *P* = (*F*_o_^2^ + 2*F*_c_^2^)/3
*wR*(*F*^2^) = 0.049	(Δ/σ)_max_ = 0.002
*S* = 0.92	Δρ_max_ = 0.89 e Å^−^^3^
2344 reflections	Δρ_min_ = −1.31 e Å^−^^3^
137 parameters	Extinction correction: none
6 restraints	Absolute structure: Flack (1983), 852 Friedel pairs
Primary atom site location: structure-invariant direct methods	Flack parameter: 0.033 (5)
Secondary atom site location: difference Fourier map	

### Special details

**Table:**

Geometry. All e.s.d.'s (except the e.s.d. in the dihedral angle between two l.s. planes) are estimated using the full covariance matrix. The cell e.s.d.'s are taken into account individually in the estimation of e.s.d.'s in distances, angles and torsion angles; correlations between e.s.d.'s in cell parameters are only used when they are defined by crystal symmetry. An approximate (isotropic) treatment of cell e.s.d.'s is used for estimating e.s.d.'s involving l.s. planes.
Refinement. Refinement of *F*^2^ against ALL reflections. The weighted *R*-factor *wR* and goodness of fit *S* are based on *F*^2^, conventional *R*-factors *R* are based on *F*, with *F* set to zero for negative *F*^2^. The threshold expression of *F*^2^ > σ(*F*^2^) is used only for calculating *R*-factors(gt) *etc*. and is not relevant to the choice of reflections for refinement. *R*-factors based on *F*^2^ are statistically about twice as large as those based on *F*, and *R*- factors based on ALL data will be even larger.

### Fractional atomic coordinates and isotropic or equivalent isotropic displacement parameters (Å^2^)

**Table:**

	*x*	*y*	*z*	*U*_iso_*/*U*_eq_	
C1	0.7832 (10)	0.7760 (5)	0.7209 (4)	0.0132 (17)	
H1	0.8193	0.7899	0.7770	0.016*	
C2	0.6807 (9)	0.6765 (5)	0.7059 (5)	0.0103 (16)	
C3	0.6386 (10)	0.6555 (6)	0.6213 (5)	0.0140 (17)	
H3	0.5718	0.5874	0.6060	0.017*	
C4	0.6952 (10)	0.7348 (6)	0.5605 (5)	0.0179 (18)	
H4	0.6661	0.7219	0.5034	0.022*	
C5	0.7939 (10)	0.8325 (6)	0.5829 (5)	0.0136 (17)	
H5	0.8333	0.8858	0.5406	0.016*	
C6	0.6217 (10)	0.5906 (6)	0.7722 (4)	0.0119 (17)	
H6	0.5072	0.5572	0.7543	0.014*	
C7	0.6751 (11)	0.4290 (6)	0.8612 (5)	0.0188 (19)	
H7A	0.7662	0.3799	0.8882	0.023*	
H7B	0.5756	0.3766	0.8474	0.023*	
C8	0.6183 (11)	0.5305 (6)	0.9193 (4)	0.0185 (19)	
H8A	0.7063	0.5453	0.9633	0.022*	
H8B	0.5063	0.5115	0.9465	0.022*	
C9	0.6006 (10)	0.6397 (6)	0.8611 (4)	0.0140 (17)	
H9A	0.4857	0.6778	0.8681	0.017*	
H9B	0.6915	0.6998	0.8735	0.017*	
C10	0.7411 (11)	0.4025 (6)	0.7151 (5)	0.023 (2)	
H10A	0.7971	0.3277	0.7324	0.034*	
H10B	0.8038	0.4368	0.6674	0.034*	
H10C	0.6210	0.3861	0.6988	0.034*	
Hg1	0.95797 (4)	1.05075 (2)	0.698176 (18)	0.01298 (8)	
I1	0.84450 (6)	1.06434 (4)	0.85667 (3)	0.01766 (13)	
I2	0.91255 (7)	1.16336 (4)	0.55341 (3)	0.02021 (14)	
N1	0.8355 (8)	0.8542 (4)	0.6627 (4)	0.0104 (14)	
N2	0.7431 (9)	0.4874 (4)	0.7847 (3)	0.0126 (15)	

### Atomic displacement parameters (Å^2^)

**Table:**

	*U*^11^	*U*^22^	*U*^33^	*U*^12^	*U*^13^	*U*^23^
C1	0.016 (4)	0.017 (3)	0.007 (4)	−0.001 (3)	−0.002 (3)	0.008 (3)
C2	0.010 (2)	0.011 (2)	0.010 (2)	0.0016 (17)	0.0016 (17)	−0.0009 (17)
C3	0.011 (4)	0.016 (3)	0.016 (5)	0.001 (3)	−0.005 (3)	−0.001 (3)
C4	0.019 (5)	0.022 (4)	0.012 (5)	0.005 (4)	−0.007 (4)	0.000 (4)
C5	0.014 (4)	0.022 (4)	0.005 (4)	0.007 (4)	−0.001 (3)	0.002 (3)
C6	0.010 (4)	0.014 (3)	0.012 (4)	0.001 (3)	−0.001 (3)	0.002 (3)
C7	0.028 (5)	0.018 (4)	0.011 (4)	0.001 (4)	0.008 (4)	0.003 (3)
C8	0.024 (5)	0.021 (4)	0.011 (4)	−0.003 (4)	0.006 (4)	−0.001 (3)
C9	0.020 (4)	0.016 (3)	0.007 (4)	0.004 (3)	0.005 (3)	−0.001 (3)
C10	0.023 (5)	0.017 (4)	0.029 (6)	−0.001 (4)	0.002 (4)	−0.006 (4)
Hg1	0.01563 (16)	0.01506 (14)	0.00825 (16)	−0.00253 (13)	−0.00074 (12)	0.00158 (13)
I1	0.0168 (3)	0.0258 (3)	0.0105 (3)	−0.0004 (2)	0.0026 (2)	−0.0031 (2)
I2	0.0254 (3)	0.0222 (3)	0.0131 (3)	−0.0003 (2)	−0.0033 (2)	0.0079 (2)
N1	0.015 (3)	0.010 (3)	0.006 (3)	−0.003 (3)	0.002 (3)	0.002 (3)
N2	0.021 (4)	0.007 (3)	0.009 (4)	−0.005 (3)	0.002 (3)	0.003 (3)

### Geometric parameters (Å, °)

**Table:**

C1—N1	1.336 (8)	C7—H7A	0.9900
C1—C2	1.384 (9)	C7—H7B	0.9900
C1—H1	0.9500	C8—C9	1.538 (9)
C2—C3	1.409 (9)	C8—H8A	0.9900
C2—C6	1.499 (9)	C8—H8B	0.9900
C3—C4	1.384 (9)	C9—H9A	0.9900
C3—H3	0.9500	C9—H9B	0.9900
C4—C5	1.376 (9)	C10—N2	1.460 (8)
C4—H4	0.9500	C10—H10A	0.9800
C5—N1	1.336 (9)	C10—H10B	0.9800
C5—H5	0.9500	C10—H10C	0.9800
C6—N2	1.498 (8)	Hg1—N2^i^	2.428 (7)
C6—C9	1.530 (9)	Hg1—N1	2.454 (5)
C6—H6	1.0000	Hg1—I2	2.6536 (5)
C7—N2	1.481 (9)	Hg1—I1	2.6819 (6)
C7—C8	1.528 (9)	N2—Hg1^ii^	2.428 (7)
			
N1—C1—C2	125.1 (7)	C7—C8—H8B	110.9
N1—C1—H1	117.4	C9—C8—H8B	110.9
C2—C1—H1	117.4	H8A—C8—H8B	108.9
C1—C2—C3	115.5 (6)	C6—C9—C8	105.5 (5)
C1—C2—C6	124.3 (7)	C6—C9—H9A	110.6
C3—C2—C6	120.1 (6)	C8—C9—H9A	110.6
C4—C3—C2	119.5 (6)	C6—C9—H9B	110.6
C4—C3—H3	120.2	C8—C9—H9B	110.6
C2—C3—H3	120.2	H9A—C9—H9B	108.8
C5—C4—C3	119.9 (7)	N2—C10—H10A	109.5
C5—C4—H4	120.0	N2—C10—H10B	109.5
C3—C4—H4	120.0	H10A—C10—H10B	109.5
N1—C5—C4	121.6 (7)	N2—C10—H10C	109.5
N1—C5—H5	119.2	H10A—C10—H10C	109.5
C4—C5—H5	119.2	H10B—C10—H10C	109.5
N2—C6—C2	113.2 (6)	N2^i^—Hg1—N1	97.59 (19)
N2—C6—C9	102.6 (5)	N2^i^—Hg1—I2	111.18 (13)
C2—C6—C9	117.3 (6)	N1—Hg1—I2	99.85 (13)
N2—C6—H6	107.7	N2^i^—Hg1—I1	102.73 (13)
C2—C6—H6	107.7	N1—Hg1—I1	98.19 (13)
C9—C6—H6	107.7	I2—Hg1—I1	138.749 (19)
N2—C7—C8	106.1 (5)	C1—N1—C5	118.2 (6)
N2—C7—H7A	110.5	C1—N1—Hg1	122.6 (4)
C8—C7—H7A	110.5	C5—N1—Hg1	118.3 (4)
N2—C7—H7B	110.5	C10—N2—C7	109.8 (5)
C8—C7—H7B	110.5	C10—N2—C6	113.0 (6)
H7A—C7—H7B	108.7	C7—N2—C6	103.1 (5)
C7—C8—C9	104.2 (6)	C10—N2—Hg1^ii^	106.5 (5)
C7—C8—H8A	110.9	C7—N2—Hg1^ii^	111.8 (5)
C9—C8—H8A	110.9	C6—N2—Hg1^ii^	112.7 (4)

Symmetry codes: (i) −*x*+2, *y*+1/2, −*z*+3/2; (ii) −*x*+2, *y*−1/2, −*z*+3/2.
